# Quizartinib (AC220) is a potent second generation class III tyrosine kinase inhibitor that displays a distinct inhibition profile against mutant-*FLT3, -PDGFRA* and -*KIT* isoforms

**DOI:** 10.1186/1476-4598-12-19

**Published:** 2013-03-07

**Authors:** Kerstin Maria Kampa-Schittenhelm, Michael Charles Heinrich, Figen Akmut, Hartmut Döhner, Konstanze Döhner, Marcus Matthias Schittenhelm

**Affiliations:** 1 Department of Hematology, Oncology, Rheumatology, Immunology and Pulmology, University Hospital Tübingen, Tübingen, Germany; 2 Department of Medicine; Division of Hematology and Medical Oncology, Portland VA Medical Center and OHSU Knight Cancer Institute, Portland, OR, USA; 3 Department of Internal Medicine III, University Hospital Ulm, Ulm, Germany

**Keywords:** AC220, Quizartinib, Leukemia, *KIT*, *FLT3*, *PDGFR*

## Abstract

**Background:**

Activating mutations of class III receptor tyrosine kinases (RTK) *FLT3, PDGFR* and *KIT* are associated with multiple human neoplasms including hematologic malignancies, for example: systemic mast cell disorders (*KIT*), non-CML myeloproliferative neoplasms (*PDGFR*) and subsets of acute leukemias (*FLT3* and *KIT*). First generation tyrosine kinase inhibitors (TKI) are rapidly being integrated into routine cancer care. However, the expanding spectrum of TK-mutations, bioavailability issues and the emerging problem of primary or secondary TKI-therapy resistance have lead to the search for novel second generation TKIs to improve target potency and to overcome resistant clones.

Quizartinib was recently demonstrated to be a selective *FLT3* inhibitor with excellent pharmacokinetics and promising *in vivo* activity in a phase II study for *FLT3* ITD + AML patients. *In vitro* kinase assays have suggested that in addition to *FLT3*, quizartinib also targets related class III RTK isoforms.

**Methods:**

Various *FLT3* or *KIT* leukemia cell lines and native blasts were used to determine the antiproliferative and proapoptotic efficacy of quizartinib. To better compare differences between the mutant kinase isoforms, we generated an isogenic BaF3 cell line expressing different *FLT3, KIT* or *BCR/ABL* isoforms. Using immunoblotting, we examined the effects of quizartinib on activation of mutant *KIT* or *FLT3* isoforms.

**Results:**

Kinase inhibition of (mutant) *KIT*, *PDGFR* and *FLT3* isoforms by quizartinib leads to potent inhibition of cellular proliferation and induction of apoptosis in *in vitro* leukemia models as well as in native leukemia blasts treated *ex vivo*. However, the sensitivity patterns vary widely depending on the underlying (mutant)-kinase isoform, with some isoforms being relatively insensitive to this agent (e.g. *FLT3* D835V and *KIT* codon D816 mutations). Evaluation of sensitivities in an isogenic cellular background confirms a direct association with the underlying mutant-TK isoform – which is further validated by immunoblotting experiments demonstrating kinase inhibition consistent with the cellular sensitivity/resistance to quizartinib.

**Conclusion:**

Quizartinib is a potent second-generation class III receptor TK-inhibitor – but specific, mutation restricted spectrum of activity may require mutation screening prior to therapy.

## Background

Gain-of-function mutations of the *FLT3*, *KIT* and *PDGFR* class III receptor tyrosine kinases (RTK) play important roles as oncogenesis-driving events in several hematologic malignancies. For example, *FLT3* mutations are predominantly found in AML associating with a poor prognosis [1-4], but are also reported in (pediatric) acute lymphoblastic leukemia (ALL) [5]. *KIT* mutations occur in the vast majority of systemic mastocytosis (SM) [6] and subsets of acute leukemia, including core-binding factor (CBF) [7] and pediatric [8] AML. Certain *FLT3* and *KIT* mutations correlate with inferior outcome in adult AML [4,9,10].

*PDGFR* mutations are frequently found in myeloproliferative disorders, such as Philadelphia chromosome-negative chronic myeloid leukemia (CML), where *PDGFR* alpha or beta fuses with another gene allowing autoactivation of the tyrosine kinase. Several fusion partners have been described, including *FIP1L1* leading to the *FIP1L1-PDGFRA* fusion gene. This translocation has been associated with hypereosinophilic syndromes and mastocytosis with eosinophilia [11-13].

Numerous tyrosine kinase inhibitors have been developed to target class III RTKs (see also Discussion). These TKIs have a variable spectrum of activity against different class III RTKs and against various mutant isoforms of these kinases. To date, translation from bench to bedside has resulted in only modest or short-lived effectiveness of these inhibitors in most entities [14-23] and only a few agents have achieved FDA-approval for selected indications such as CML and HES. With the exception of Ph+ALL, no TKIs have been approved for treatment of acute leukemia so far.

Quizartinib is a novel second generation class III receptor tyrosine kinase inhibitor with superior pharmaceutical properties and an excellent pharmacokinetic profile compared to other agents. Quizartinib was demonstrated to have high efficacy and tolerability in tumor xenograft models that express a *FLT3* ITD mutant kinase [24,25].

A previous study used recombinant enzyme in *in vitro* kinase assays to identify that quizartinib targets related class III RTKs, such as wildtype and gain-of-function mutant *KIT* and *PDGFR* isoforms [24].

Using several cell based assays, we now show, that quizartinib treatment of leukemic cells leads to inhibition of mutant *KIT*, *PDGFR* and *FLT3* isoforms - with resultant inhibition of cellular proliferation and induction of apoptosis*.* These effects are seen *in vitro* as well as *ex vivo* (primary leukemic blasts). Importantly, potent antitumor activity was seen against distinct (mutated) kinase isoforms, including *FIP1L1-PDGFRA*, and *FLT3* ITD, *FLT3* TKD1 and *FLT3* TKD2 mutations. Whereas some mutant-*KIT* and –*FLT3* isoforms were sensitive to quizartinib treatment, some mutations such as *FLT3* D835V and the most prevalent *KIT* gain-of-function mutation detected in CBF AML, *KIT* D816V, was relatively insensitive with regard to quizartinib treatment.

Quizartinib is currently under clinical investigation in *FLT3* ITD and wildtype AML. Our data suggests that quizartinib may be an attractive agent for clinical investigation in other settings as outlined here. This would not include the group of mutant-*KIT* CBF AML that have *KIT* D816V mutations. However, patients with CBF AML with *KIT* D816Y or exon 11 mutations or patients with solid tumors associated with *KIT* and *PDGFR* mutations, such as GIST might benefit from this agent. Clinical mutation analysis could help identify individuals that are the most likely to respond to quizartinib.

## Results

### Quizartinib inhibits cellular proliferation of mutant-*FLT3*, -*KIT* or –*PDGFRA* leukemia cell lines in a dose dependent manner

Quizartinib was previously reported to be a potent inhibitor of wildtype *FLT3* and *FLT3* ITD kinases [24]. Structural considerations suggest quizartinib could inhibit other members of the class III RTK family that are frequently mutated in leukemia or myeloproliferative disorders (i.e. *KIT* and *PDGFR*). These findings prompted us to evaluate quizartinib sensitivity in a variety of leukemia cell line models harboring RTK mutations.

The human mast cell leukemia cell lines HMC1.1 (*KIT* V560G) and HMC1.2 (*KIT* V560G + D816V), the murine mast cell line p815 (harboring a *KIT* D814Y mutation analogous to the human D816Y mutation), the eosinophilic leukemia cell line EOL-1 (*FIP1L1-PDGFRA*), the CBF AML cell line Kasumi-1 (N822K), the myeloid leukemia cell line MOLM14 (heterozygous *FLT3* ITD), M-07e (growth factor dependent wildtype *KIT*), the APL cell line HL60 (growth factor independent, wildtype *FLT3* and *KIT*), the lymphoblastic leukemia cell line Jurkat (no known activated RTK) and the CML blast crisis cell line K562 (BCR/ABL1) were treated with quizartinib in a dose-dependent manner for 48 hours and the cellular antiproliferative capacity was measured using an XTT-based assay.

**Figure 1:**
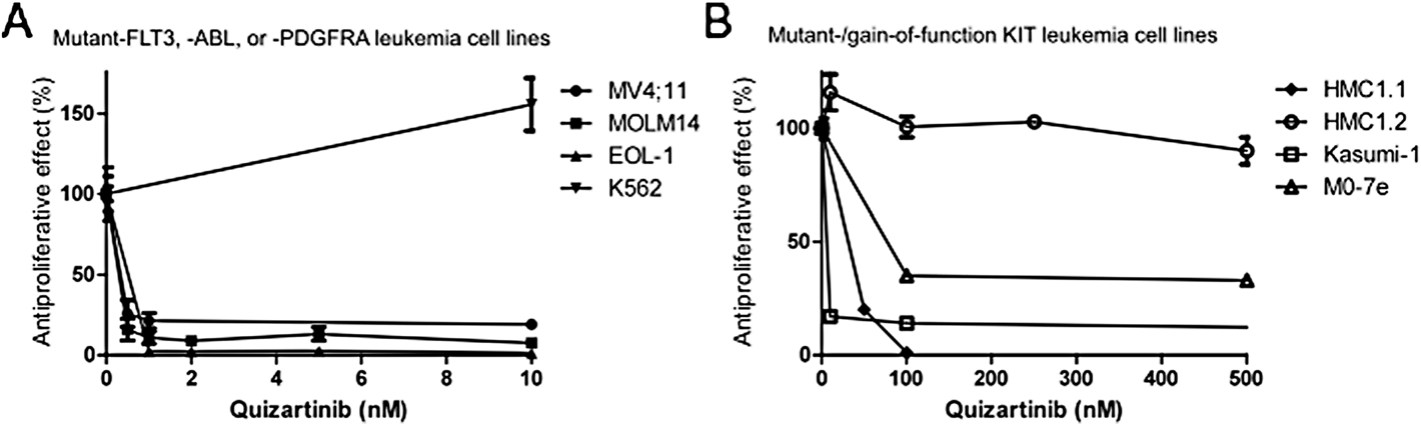
**Quizartinib inhibits cellular proliferation of *KIT*-, *FLT3*- or PDGFR-dependent leukemia cells.** Dilution series of quizartinib for several cell lines harboring mutant-*FLT3*, *ABL1* or *PDGFRA* (**1A**) or mutant-*KIT* isoforms (**1B**) were performed and cellular proliferation was assessed using an XTT-based assay. Sensitivity towards quizartinib varied widely between the tested cell lines. IC50s and mutation isoforms for all tested cell lines are listed in Table 1.

The proliferation of cell lines with *FLT3* ITD (MV4;11, MOLM14), *FIP1L1-PDGFRA* (EOL-1), ligand-stimulated wild-type *KIT* (M0-7e), or certain *KIT* mutations (*KIT* exon 11 V560G, HMC1.1; Kasumi *KIT* exon 17 N822K) was strongly inhibited by quizartinib (Figure 1). In contrast, the proliferation of a cell line with a *KIT* exon 11 V560G and *KIT* exon 17 D816V mutation on the same allele (HMC1.2) was basically insensitive to quizartinib. Noteworthy, the murine cell line p815, harboring an alternative (tyrosine) substitution at the same codon (*KIT* D814Y, which corresponds to D816Y in human *KIT*), retained intermediate sensitivity to quizartinib.

As experimental controls, we also tested quizartinib against several cell lines lacking an activated type III RTK (K562 [*BCR-ABL1*], Jurkat [no known tyrosine kinase oncogene], and HL60 [no known tyrosine kinase oncogene, but expressed wild-type *FLT3*]; quizartinib had no meaningful anti-proliferative effects against these cell lines (IC50 > 10,000 nM; Table 1).

**Table 1:** Non-linear regression analysis of IC50s (Antiproliferation)

**Cell line**	**Target**	**IC50 (nM)**
		***Inhibition of proliferation***
HMC1.1	*KIT* V560G	14
HMC1.2	*KIT* V560G/D816V	1727
HMC1.2, 0.5% FBS	*KIT* V560G/D816V	263
p815	*KIT* D814Y (murine)	445
Kasumi-1	*KIT* N822K	36
M-07e + SCF	*KIT*-activated	77
M-07e + GM-CSF	*GM-CSF* signaling	not reached*
EOL-1	*FIP1L1-PDGFRA*	1
K562	*BCR/ABL*	not reached*
HL60	unknown	not reached*
Jurkat	unknown	not reached*
MV4;11	*FLT3* ITD (hemizygous)	< 1
MOLM14	*FLT3* ITD	< 1
MOLM14 + DMSO	*FLT3* ITD	not reached*
Pat.221	CBF AML (*KIT* WT)	675
Pat.279	CBF AML (*KIT* WT) / *FLT3* amplification (subclone)?	3434
Pat.299	CBF AML (*KIT* WT)	7248
Pat.305	CBF AML (*KIT* WT)	7079
Pat.375	CBF AML (*KIT* N/A)	503
Pat.379	CBF AML (*KIT* WT)	806
Pat.368	*FLT3* amplification ?	2700
Pat.601	*FLT3* ITD	1153
Pat.176	*FLT3* ITD (Beta1)	not reached*
Pat.602	*FLT3* ITD (Beta1)	not reached*

* tested up to 10 000 nM.

The table summarizes estimated IC50 values obtained by non-linear regression analysis for the antiproliferative activity of quizartinib in leukemia cell lines and primary native leukemia blasts.

The cell line HMC1.2 was additionally pre-treated with reduced serum (0.5% FBS) to address the influence of methodology aspects on sensitivity profiles. To exclude solvent-associated non-specific cytotoxicity, the MOLM-14 cell line was treated with DMSO using the highest concentration for the quizartinib dose experiments.

DMSO alone, used in the highest concentration in any of the quizartinib dosing experiments, had no significant antiproliferative effect (MOLM14 cell line, Table 1).

To further show the specificity of the anti-proliferative effect of quizartinib, we tested the effects of this drug against M-07e cultured in GM-CSF rather than SCF: Whereas the SCF-stimulated M-07e were sensitive to quizartinib (IC50 77 nM), GM-CSF stimulated M-07e were completely resistant (quizartinib IC50 > 10,000 nM).

Notably, the sensitivity patterns observed for different mutant RTK isoforms were not totally consistent with IC50s previously published in a kinase assay reported by Zarrinkar and colleagues [24] (e.g. IC50_KIT D816V_ 150 nM versus >1500 nM in our assays). There are several possible explanations for this disparity. First, the results by Zarrinkar et al. were performed using drug binding to recombinant kinases as opposed to enzymatic inhibition of full-length cellular kinases. Second, while we did not change FBS levels prior to quizartinib administration (10% for leukemia cell lines, 20% for native leukemia blasts) – the earlier report by Zarrinkar et al. [24] used cells that were pre-sensitized by exposure to reduced serum levels (0.5% FBS) 12 hours prior to therapy.

Serum deprivation is a commonly used method to reduce serum-drug interactions – but also has profound cellular effects including accumulation and synchronization of cells in the G1/G0 [26]. Moreover, given the high protein binding of quizartinib (99% protein bound), it is not surprising that changes in serum concentration would affect drug potency in cell-based models.

For example, we repeated our experiments with the *KIT* D816V-positive cell line HMC1.2, but this time cultured the cells in serum-reduced media overnight prior to quizartinib treatment the next day. This change in experimental conditions profoundly altered the antiproliferative effect of quizartinib, as the IC50 for serum-deprived cells was ~260 nM compared to the serum replete conditions (IC50 ~1700 nM). The potency of the serum-deprived cells is in the range of the predicted IC50 for the D816V mutation reported by Zarrinkar et al. [24] (Table 1).

This effect was further validated using an isogenic cell model as well as native *FLT3* ITD positive leukemia blasts as described below. (Please, refer to Table 2 and Additional file 1: Figure S1 for similar experiments using native cells and to Table 3 for serum-deprivation experiments using Ba/B3 FLT3 ITD and KIT D816V cells).

**Table 2:** Non-linear regression analysis of IC50s (Apoptosis/Viability)

** Cell line**	** Target**	**IC50 (nM) **
		***Induction of apoptosis***
HMC1.1	*KIT* V560G	31
HMC1.2	*KIT* V560G/D816V	not reached*
p815	*KIT* D814Y (murine)	341
Kasumi-1	*KIT* N822K	67
M-07e + SCF	*KIT*-activated	78
M-07e + GM-CSF	unspecific stimulation	not reached*
EOL-1	*FIP1L1-PDGFRA*	< 1
K562	*BCR/ABL*	not reached*
HL60	N/A	not reached*
Jurkat	N/A	not reached*
MV4;11	*FLT3* ITD (hemizygous)	2
MOLM14	*FLT3* ITD	3
GIST822	*KIT* K642E	109
GIST48	*KIT* V560D/D820A	not reached*
Pat.368	*FLT3* amplification ?	2998
Pat.601	*FLT3* ITD	876
Pat.695, 20% FBS	*FLT3* ITD	2335
Pat.695, 0.5% FBS	*FLT3* ITD	25
Pat.139, 20% FBS	*FLT3* ITD, *Relaps*	760
Pat.139, 0.5% FBS	*FLT3* ITD, *Relaps*	10
		*Reduction of viable cells*
Pat.507	CBF AML (*KIT* WT)	1275
Pat.317	CBF AML (*KIT* D816Y)	1294
Pat.521	CBF AML (*KIT* WT)	2018
Pat.305	CBF AML (*KIT* WT)	2954
Pat.511	CBF AML (*KIT* WT)	4272
Pat.281	CBF AML (*KIT* WT)	5758
Pat.279	CBF AML (*KIT* WT) / *FLT3* amplification? (subclone)	6607
Pat.523	CBF AML (*KIT* WT)	7175
Pat.361	CBF AML (*KIT* D816V)	8443
Pat.239	CBF AML (*KIT* D816V)	not reached*

* tested up to 10 000 nM.

The table summarizes estimated IC50 values obtained by non-linear regression analysis for the cytotoxic activity of quizartinib in leukemia cell lines and primary native leukemia blasts. Native patient blasts were cultured in 20% FBS." to "Cell lines were cultured in 10% FBS; native patient blasts were cultured in 20% FBS. To address methodology aspects towards sensitivity profiles two native FLT3 ITD + patient samples (Pat. 695 with newly diagnosed AML, Pat. 139 with relapsed AML) were co-treated with reduced serum (0.5% FBS).

In addition IC50 estimates for the proapoptotic effect of quizartinib in the imatinib-sensitive GIST solid tumor cell line GIST882, harboring a K642E mutation, and the imatinib-insensitive cell line GIST48, harboring a V560D mutation in addition to a D820A mutation in the tyrosine kinase domain, are provided, revealing sensitivity profiles similar to imatinib.

**Table 3:** Estimated IC50s for the proapoptotic and antiproliferative effects of quizartinib in an isogenic cell model of Ba/F3 cells transfected with various mutant TKs

**Isoform**	**Mutation locus**	**IC50 (nM)**	**IC50 (nM)**	**IC50 (nM)**	**IC50 (nM)**
		***Ba/F3 transfectants***		***leukemia cell lines***	
		***Inhibition of proliferation***	***Induction of apotosis***	***Inhibition of proliferation***	***Induction of apotosis***
*BCR/ABL*	fusion	n.r.*	n.r.*	not reached *(K562)*	n.r.* *(K562)*
*FLT3 WT*	N/A	49	11		
*FLT3 ITD, 10% FBS*	juxtamembrane domain	9	5	<1 *(MOLM14)*	3 *(MOLM14)*
*FLT3 ITD, 0.5%FBS*	juxtamembrane domain	<1	N/D		
*FLT3 K663Q*	tyrosine kinase domain I	14	23		
*FLT D835V*	tyrosine kinase domain II	172	888		
*FLT3 D835Y*	tyrosine kinase domain II	84	24		
*KIT WT*	N/A	474	n.r.*	77 *(M0-7e)*	210 *(MO-7e)*
*KIT D816F*	tyrosine kinase domain II	2871	6254		
*KIT D816V, 10% FBS*	tyrosine kinase domain II	3074	8982	1727 *(HMC1.2)*	n.r.* *(HMC1.2)*
*KIT D816V, 0.5% FBS*	tyrosine kinase domain II	633	N/A		
*KIT D816Y, 0.5% FBS*	tyrosine kinase domain II	366	611	*445 (p815)*	341 *(p815)*
parental	N/A	n.r.*	n.r.*		
parental + DMSO	N/A	n.r.*	n.r.*		

*(not reached with tested doses up to 10 000 nM).

Sensitivity of quizartinib is distinct to and differs widely in between different tyrosine kinase isoforms transfected into an isogenic Ba/F3 cellular background. Estimated IC50s were computed using non-linear regression analysis of an average mean of at least 3 experiments for each cell line.

If applicable, IC50s of leukemic cell lines harboring a similar mutation are provided (rows on the right).

Influence of serum-deprivation on sensitivity profiles of quizartinib was tested in two cell strains (Ba/F3 *FLT3* ITD or *KIT* D816V): Cells were cultured in media with a reduced serum concentration (0.5% FBS) and treated with quizartinib the next day.

Solvent-associated non-specific cytotoxicity was excluded using the parental Ba/F3 cell strain treated with DMSO in the highest concentration used for the quizartinib dose experiments.

### Quizartinib induces apoptosis in *in vitro* leukemia cell lines

The extraordinary antiproliferative effect seen in some cell models tested, was also accompanied by microscopically condensed pyknotic cells that accumulated over time. This observation suggests that quizartinib may induce apoptosis via inhibition of (mutant) *FLT3*, *KIT* or *PDGFRA*.

Using an annexin V-based immunofluorescence assay, we were able to demonstrate potent dose-dependent induction of apoptosis in several leukemia cell lines:

In analogy to the demonstrated antiproliferative effects, evaluation of quizartinib in several cell lines lacking an activated type III RTK (K562, Jurkat and HL60) did not reveal any significant proapoptotic effects. In contrast, cell lines harboring *FLT3* ITD (MV4;11, MOLM14), *FIP1L1-PDGFRA* (EOL-1), SCF (but not GM-CSF)-stimulated wild-type *KIT* (M0-7e), or certain *KIT* mutations (*KIT* exon 11 V560G, HMC1.1; Kasumi *KIT* exon 17 N822K) potently underwent apoptosis upon quizartinib exposure with IC50s in the lower nanomolar ranges (Table 2). Notably, IC50s were similar or somewhat higher compared to the antiproliferative effects achieved in these cell lines (compare with Table 1).

**Figure 2:**
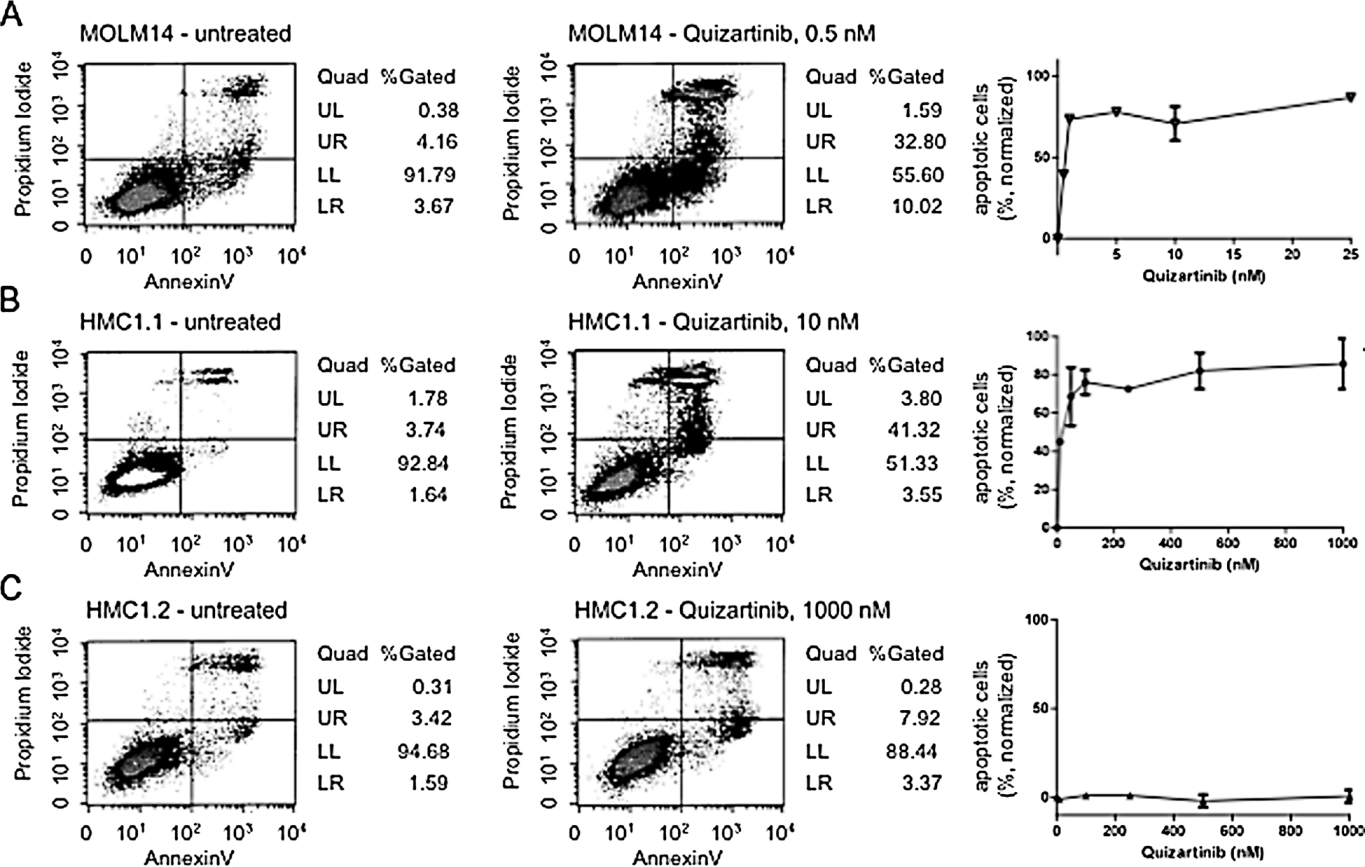
**Quizartinib potently induces apoptosis in selected leukemia cell line models.** Leukemia cell lines harboring *FLT3*, *KIT* or *PDGFRA* mutations were treated with various doses of quizartinib for 48 hours and induction of apoptosis was measured using an annexin V-based assay. Representative density plots indicating early phase apoptosis (annexin V-positive cells) or late phase apoptotic cells (propidium iodide positivity) fractions are shown in a quadrant density dot plot. Whereas the *FLT3* ITD + cell line MOLM14 (**A**) and the mutant-*KIT* V560G + cell line HMC1.1 (**B**) reveal potent sensitivity in the lower nanomolar range, the sister cell line HMC1.2 (**C**), harboring an additional *KIT* D816V mutation, is insensitive towards quizartinib treatment. Data presented in the dose–response curves on the right represents the average mean of at least 3 separate experiments (each normalized to the untreated controls, which are set to zero). IC50s of all tested cell lines are provided in Table 2.

HMC1.2, the sister cell line of HMC1.1 harboring an additional *KIT* D816V mutation, revealed a complete loss of sensitivity towards quizartinib in all tested doses (Figure 2; IC50s are provided with Table 2). This finding suggests that the distinct mutant-*KIT* isoform directly orchestrates sensitivity towards quizartinib. In this context it is noteworthy, that the *KIT* D814Y-positive (D816Y in human *KIT*) murine cell line p815 was still capable to induce apoptosis with an IC50 in the hundreds nanomolar range (Figure 2).

### Comparison of quizartinib sensitivity towards different leukemia-driving *KIT* and *FLT3* mutations in an isogenic cellular background

Quizartinib potently inhibits cellular proliferation and induces apoptosis in leukemia cell lines that are dependent on *FLT3*, *KIT* or *PDGFRA* activity. However, the potency of quizartinib differs widely between the tested cell lines – from complete insensitivity to doses in the low nanomolar range.

The divergent inhibitory effects may be due to differential sensitivity profiles of different (mutant) RTK isoforms (compare findings about HMC1.1 versus HMC1.2 cells) – but may also have been obscured by additional genomic abnormalities contributing to leukemogenesis and resistance to therapeutics.

To exclude cell line-specific off-target biology interfering with the effects of kinase-inhibition, we tested leukemia-driving RTK mutations in an isogenic cellular background: Various human (mutant) *FLT3* or *KIT* isoforms were stably transfected in the IL3-dependent murine pro B-cell line Ba/F3. Activation of the transfected mutant isoforms was demonstrated by selecting for cells with IL-3 growth factor-independent proliferation. However, BaF3 cells expressing wildtype KIT or FLT3 isoforms required the addition of the corresponding ligand, (*KIT* (SCF) or *FLT3* (*FLT3*L)).

**Figure 3:**
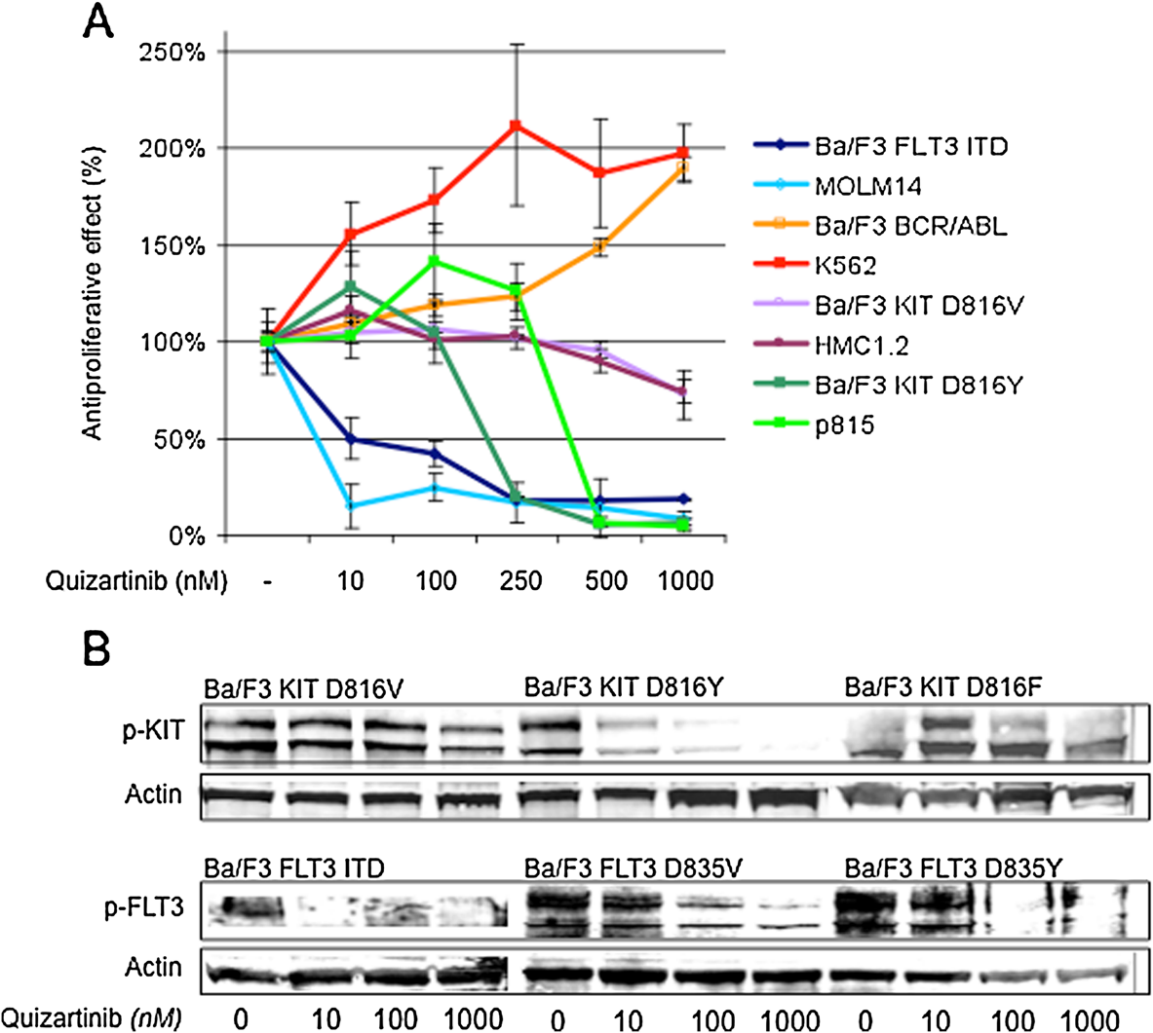
**Cellular effects of quizartinib are tyrosine kinase-mediated (A) Quizartinib displays distinct antiproliferative effects of genetically altered Ba/F3 cells in dependence of the tyrosine kinase isoform transfected.** The sensitivity of inhibition of proliferation is thereby similar to the sensitivity achieved in natural leukemia cell lines harboring a similar mutation. Estimated IC50s are provided in Table 3 along with IC50s for the proapoptotic effects in the same cellular context. (**B**) The observed cellular effects are directly linked to the potency of inhibition of phosphorylation of mutant-*KIT* and *FLT3* isoforms. Whole cell lysates of Ba/F3 cells transfected with different human mutant-*KIT* or -*FLT3* isoforms were immunoblotted using a pan-phosphotyrosine antibody or a total-*KIT* or -*FLT3* antibody. Pretreatment of cells with quizartinib revealed isoform-specific inhibition of phosphorylation. Notably, inhibition of phosphorylation of the D816V mutation was significantly reduced compared to the D816Y and D816F isoforms.

We were able to directly cross-check the clinically most relevant RTK mutations in acute leukemia (i.e. *FLT3*-ITD, *KIT* D816V/Y, *BCR*/*ABL1*) transfected into an isogenic Ba/F3 background against a panel of leukemic cell lines harboring a corresponding RTK mutation. Comparison of inhibition of cellular proliferation after quizartinib treatment revealed strong correlation between naturally occurring and engineered cell lines expressing identical mutant kinases (Figure 3A): Ba/F3 cells stably transfected with a vector encoding for a *FLT3* ITD were equally highly sensitive to quizartinib compared to the human *FLT3* ITD positive leukemia cell line MOLM14. Vice versa, transfection of a *KIT* D816V mutation retained Ba/F3 cells highly resistant towards quizartinib, which is consistent with findings in the human mast cell leukemia cell line HMC1.2 as discussed earlier. Interestingly, replacing the valine substitution with tyrosine at codon 816 (D816Y) rendered Ba/F3 cells to relative sensitivity to quizartinib – so was the *KIT* D814Y-positive cell line p815.

This observation is not unique to quizartinib – but is in line with previous data for other *KIT* tyrosine kinase inhibitors, such as dasatinib [27]. In this context, a recent study suggested structural reasons that underlay drug sensitivity of different mutant-*KIT* kinases using sunitinib and imatinib mesylate [28].

Surprisingly, transfection of *BCR/ABL1* into Ba/F3 cells not only did not halt proliferation of cells – but did confer a proliferation advantage for quizartinib treated cells in a dose-dependent manner. This observation deserves further exploration with regard to molecular mechanisms.

Together, these findings suggest a direct mutant-specific tyrosine kinase-mediated effect of quizartinib towards modulation of cellular proliferation. Table 3 provides additional information of sensitivity patterns, with regard to inhibition of proliferation as well as induction of apoptosis, for several mutant-*FLT3*, -*KIT* and *BCR/ABL1* isoforms transfected into an isogenic Ba/F3 cellular background: Of note, transfection of a *FLT3* D835V kinase domain mutation, which is homologous to D816V in *KIT*, reveals restricted sensitivity towards quizartinib – which is in line with a recent study by Smith and colleagues demonstrating a conformational clash preventing proper binding of quizartinib to the FLT3 binding pocket [29]. Importantly, our data further show that alternative substitution of aspartic acid with a tyrosine residue (D835Y) renders cells to sensitivity, which underlines our findings for *KIT* D816Y as discussed above.

### Inhibition of cellular proliferation associates with distinct inhibition of phosphorylation of the target receptor tyrosine kinase in an isogenic cell model

In our cell biology experiments, the sensitivity of the tested cell lines to quizartinib was linked to inhibition of (mutant) class III RTK. The isogenic cell models confirm IC50s obtained for the leukemia cell lines harboring similar mutations, further suggesting a direct interaction of tyrosine kinase inhibition and the observed antiproliferative and proapoptotic effects in the tested cell lines (rather than off-target effects).

To address this question at the protein level, we additionally performed immunoblotting experiments for Ba/F3 cell lines transfected with mutant *KIT* or *FLT3* kinases and treated with quizartinib for 90 minutes. Indeed, sensitivity towards quizartinib, as indicated by loss of RTK autophosphorylation, proved to be kinase-specific and was in agreement with the functional assays using the same cell lines. Notably, the *KIT* D816V mutation, exchanging aspartic acid for valine at codon 816 and thus rendering the kinase autophosphorylated, does not show significant reduction of phosphorylation levels – while tyrosine or phenylalanine substitutions at the same codon (D816Y or D816F), similarly leading to autoactivity of the kinase, proof to be sensitive towards quizartinib treatment with loss of autophosphorylation in the nanomolar ranges (Figure 3B). Interestingly, D816Y was thereby dephosphorylated at the glycosylated membrane-bound (~145 KDa), as well as the intracellular isoform (~125 KDa) – whereas sensitivity of D816F was virtually restricted to the glycosylated isoform.

These results are in line with the viability assays provided in Figure 3A and Table 3, and again argue against nonspecific off-target – but for TK-mediated effects. Moreover, it underlines that TKI sensitivity patterns are not just tyrosine kinase, kinase-domain or codon specific – but may even depend on the type of amino acid substitution at a given codon.

### *In vitro* inhibition of cellular proliferation by quizartinib translates into *ex vivo* antiproliferative effects in native leukemia blasts

We further evaluated the antiproliferative effects of quizartinib using native blasts isolated from patients with newly diagnosed *FLT3-* or *KIT-*activated AML (additional patient characteristics are provided in Additional file 2: Table S1 with the online version of the article).

Notably, quizartinib was able to inhibit proliferation of *ex vivo* CBF AML blasts and *FLT3* ITD positive blasts in the upper nanomolar or lower micromolar ranges (Table 1).

CBF AML is associated with high CD117 (i.e. *KIT*) expression and/or autoactivating mutations within the *KIT* gene [7]. *KIT* mutation screening of exons 8, 9, 11, 13 and 17 was performed. No autoactivating mutation in our patient cohort used for antiproliferation assays was detected, suggesting a paracrine activation of *KIT* in the quizartinib-responsive patient cohort as demonstrated earlier for ~50% of *FLT3*/*KIT* wildtype patients [30].

In *FLT3*-associated leukemia patients, the antiproliferative effect of quizartinib was inconsistent – with refractory as well as sensitive cases identified. For example, one case sensitive to quizartinib treatment was from a patient with an *MLLT3*-*MLL* rearrangement (patient #368). We were not able to detect any *FLT3* or *KIT* mutations in this patient – although, karyotyping revealed trisomy of chromosome 13 (*FLT3* genomic location), which potentially contributed to treatment response via *FLT3* amplification. Potent inhibition of amplified *FLT3* wildtype gene via quizartinib was recently shown in an *in vitro* leukemia cell model using the SEM-K2 ALL cell line by Gunawardane and colleagues [31]. Another of our cases demonstrating sensitivity towards quizartinib harbored a *FLT3* ITD, but interestingly, two more cases with a *FLT3* ITD were refractory to quizartinib (patient #176 and #602).

*FLT3* ITD sequencing revealed that the internal tandem duplication was located in the beta1 sheet of the first tyrosine kinase domain in both resistant cases. This particular class of mutant kinase is resistant to *FLT3* inhibition by midostaurin (PKC412) and is associated with a poor clinical outcome [32-34]. Our data suggests a similar sensitivity profile for quizartinib against *FLT3* ITD-beta1 mutant kinases.

### Quizartinib induces apoptosis in *ex vivo* native leukemia cells

We next tested isolated native blasts derived from patients with newly diagnosed AML to confirm the proapoptotic effect observed for quizartinib in *in vitro* leukemia and isogenic mutant-TK models (IC50s for all patients are provided with Table 2; patient characteristics are available as supplementary material in Additional file 2: Table S1).

One sample, taken from a bone marrow aspirate of a patient with *de novo* AML, was identified to harbor a *FLT3* ITD mutation in the juxtamembrane domain of the gene (patient #601). IC50 was in the higher nanomolar range – which is considerably higher compared to the *in vitro FLT3* ITD models. The cause of this discrepancy is unknown, but is commonly observed in *ex vivo* blasts compared to *in vitro* models [35,36]. In addition to the above comments about the effect of serum concentration on sensitivity to quizartinib, other genomic abnormalities acquired in the context of complex cytogenetic AML may have contributed to the observed effects in cultured *ex vivo* blasts.

In a second patient sample, obtained from the bone marrow aspirate of an elderly patient with *MLL*-associated AML – including trisomy of chromosome 13 (leading to overrepresentation of the *FLT3* gene), quizartinib treatment induced apoptosis in these cells with an IC50 ~3000 nM. Due to the high bioavailability of quizartinib, *ex vivo* IC50s in the lower micromolar ranges may translate into antileukemic activity *in vivo* – but this observation needs clinical validation.

**Figure 4:**
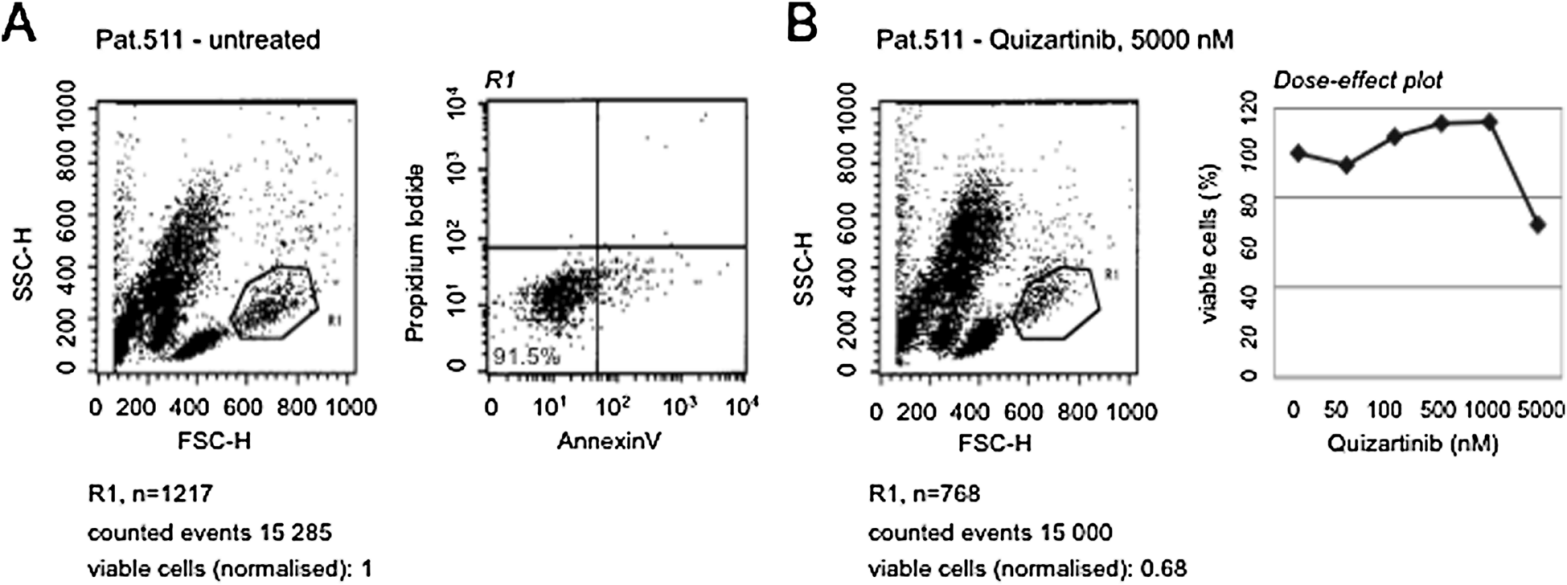
**Quizartinib reduces the proportion of viable cells in samples of native leukemia cells.** Patient samples were cultured in DMEM containing 20% FBS and treated with different concentrations of quizartinib for 48 hours. (**A**) The plot of one patient is shown. The mononuclear cell fraction was gated in a FSC/SSC scatter and viability was confirmed using an annexin V/propidium iodide (PI) stain demonstrating negativity for annexin V and PI. (**B**) Relative reduction of the proportion of viable cells in response to quizartinib was assessed by flow cytometry. A dose-effect plot of all tested concentrations of quizartinib is shown on the right. Estimated IC50s by non-linear regression analysis of all tested patient samples are provided in Table 2.

A subset of native leukemia samples analyzed presented with a high proportion of dead/apoptotic cells in the untreated control samples. This obscured conventional analysis via annexin/PI staining of proapoptotic effects induced by quizartinib treatment. Nevertheless, we were able to assess reduction of the proportion of viable cells in a cohort of CBF AML patients 48 hours after quizartinib treatment compared to treatment-naive cells (Figure 4 for representative analysis of one patient). Table 2 provides a summary of IC50s of all tested samples.

The cytotoxic effect of quizartinib varied widely. Analysis of the *KIT* mutation status revealed a wildtype gene for *KIT* in most patients – some of these were sensitive to quizartinib therapy, arguing for paracrine activation of *KIT or FLT3*[30]*.*

Of interest, similar to our cell line results, two patients with a *KIT* D816V mutation revealed relative insensitivity towards quizartinib. However, as predicted by our *in vitro* models (see also Figure 3), one patient with CBF AML harboring the less common *KIT* D816Y mutation demonstrated sensitivity with an IC50 for reduction of viable cells of approximately 1300 nM. Notably, this lies in the same range as seen for the proapoptotic effect of *ex vivo* blasts of a newly diagnosed treatment-naive patient (patient #601) harboring a *FLT3* ITD (JM) mutation. Additional patient characteristics are provided in Additional file 2: Table S1 with the online version of the article.

In general, the reported IC50s in our study for native leukemia cells lie in contrast to a previous report by Zarrinkar and colleagues [24], which have suggested IC50s for *FLT3* ITD-positive native blasts in the lower nanomolar ranges.

Several issues need to be discussed in this context. Besides individual cell-context specific additional effects (such as additional mutations rendering signal transduction pathways or drug sensitivities), which may have obscured TK-targeted effects of quizartinib, a couple of methodology-related aspects need to be addressed: As discussed earlier, we did not use serum reduced conditions in our assays – but serum-rich media containing 10% FBS for cell lines and 20% FBS for experiments involving native blasts. Moreover, it has been reported that blasts obtained from patients with relapsed *FLT3* ITD-positive leukemia may show higher sensitivities towards tyrosine kinase inhibitors due to a higher addiction to *FLT3* gain-of-function signal transduction of leukemia blasts in the relapse setting compared to d*e novo* AML samples [37]. Notably, the Zarrinkar study evaluated the efficacy towards quizartinib in relapsed patients while our work included only specimens from newly diagnosed patients. We addressed these issues and treated a newly diagnosed patient with AML as well as a patient with relapsed AML, both harboring a FLT3 ITD mutation, with quizartinib in a dose-dependent manner. Both samples were cultured in serum-repleted (20% FBS) as well as serum-reduced (0.5% FBS) conditions. In line with our theory, the average concentration to induce apoptosis was markedly reduced with IC50s in the low nanomolar range in samples cultured in serum-reduced conditions. Even more, sensitivity towards quizartinib was increased in the relapsed leukemia patient. Exemplary AnnexinV-based density plots illustrating the influence of culture conditions with regard to the achievable proapoptotic effects (~22% versus 80% dead/apoptotic cells upon exposure to 10 nM quizartinib when cultured in 20%, resp. 0.5% FBS) are provided as Additional file 1: Figure S1; IC50s are provided with Table 2.

### Antitumor activity of quizartinib in mutant-*KIT* solid tumor cell lines

Besides acute leukemia, *KIT* mutations are found in a large proportion of gastrointestinal stromal tumors (GIST) [38], in subsets of seminomas [39] and melanoma [40]. PDGFR mutations are further reported in myeloproliferative disorders and GIST as well [38].

KIT or PDGFRA tyrosine kinase inhibition is the only known medical treatment option for advanced GIST. Due to the excellent bioavailability properties of quizartinib, higher plasma concentrations are achieved compared to other inhibitors with a similar sensitivity profile. This may be advantageous in particular for the treatment of solid tumor lesions.

**Figure 5:**
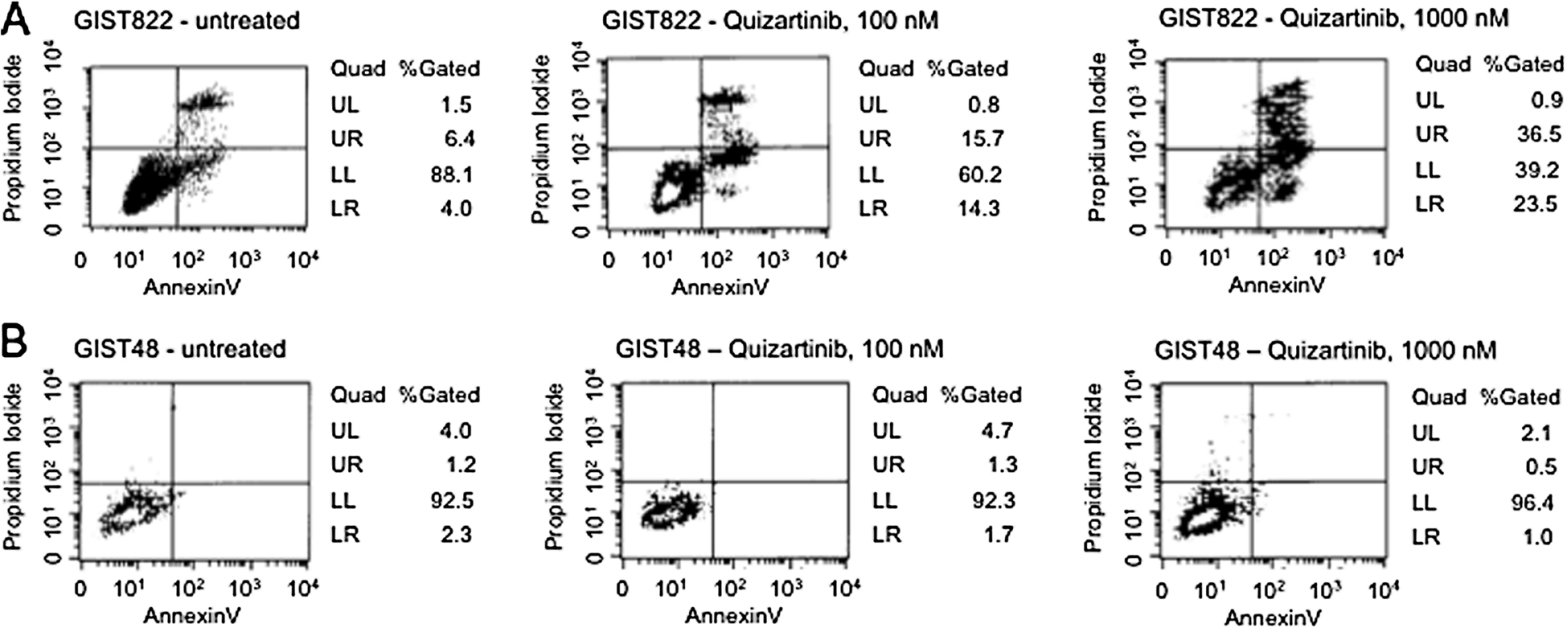
**Quizartinib selectively induces apoptosis in gastrointestinal stromal tumor (GIST) cells.** The imatinib-sensitive cell line GIST822, harboring a KIT K642E mutation (**A**) and the imatinib-insensitive cell line GIST48, harboring a KIT V560D plus an additional D820A mutation, were treated with quizartinib for 7 days and assayed for induction of apoptosis. Quadrant density dot plots are provided showing the apoptotic cell fraction as indicated by annexin V positivity (LR quadrant: early apoptosis) and/or positive propidium iodide stain (UR: late apoptosis, UL: dead cells) of quizartinib-treated versus treatment-naive cells. Estimated IC50s by non-linear dose regression analysis are provided with Table 2.

We treated an imatinib-sensitive GIST cell line (GIST822) harboring a *KIT* exon 13 mutation (K642E) and a second cell line, GIST48, harboring an imatinib-sensitive V560D mutation plus a secondary imatinib-insensitive activation loop mutation (D820A) with varying concentrations of quizartinib. Due to a much slower in vitro cell doubling time of the GIST cell lines compared to leukemic cell lines, GIST cells were treated for 7 days. Figure 5 demonstrates a potent proapoptotic effect of quizartinib targeting the KIT K642E mutation in the GIST822 cell line, whereas the imatinib-insensitive cell line GIST48 did not display any significant signs of induction of apoptosis following quizartinib treatment. Calculated IC50s are provided in Table 2.

## Discussion

Tyrosine kinase inhibitors are rapidly entering into the clinic. These agents are subject of intensive clinical investigation for the treatment of acute leukemia. For example the following inhibitors are under investigation for mutant *ABL1*, *FLT3* or *KIT*-associated subtypes (clinicaltrials.gov): sorafenib (e.g. AML: NCT00217646, NCT00373373), sunitinib (e.g. AML: NCT00783653), dasatinib (e.g. CBF-AML: NCT00850382; SM: NCT00979160; Ph + ALL: NCT00103701, NCT00940524), imatinib (e.g. AML: NCT00707408, NCT00744081; activated RTKs/various tumors: NCT00171912), lestaurtinib (e.g. AML: NCT00030186, NCT00079482), tandutinib (e.g. AML: NCT00064584, NCT00274248), masitinib (e.g. SM: NCT00814073) and nilotinib (e.g. AML: NCT01222143; SM/HES: NCT00109707).

Quizartinib (formerly AC220), a N-(5-tert-butyl-isoxazol-3-yl)-N’-{4-[7-(2-morpholin-4-yl-ethoxy)imidazo [2,1-b] [1,3] benzothiazol 2-yl]phenyl}urea dihydrochloride, is a novel class III tyrosine kinase inhibitor with promising *in vitro* as well as i*n vivo* activity against *FLT3* wildtype and mutant isoforms [24]. Compared to other tyrosine kinase inhibitors in evaluation for the treatment of acute leukemia subtypes, quizartinib provides superior bioavailability with longer and higher plasma concentrations achieved *in vivo*[25] thereby targeting and suppressing the activated kinases more effectively.

Early data from a phase II trial of quizartinib in refractory or relapsed *FLT3* ITD + patients revealed an acceptable safety profile. Interim analysis of 62 patients (29 female, 33 male) was previously presented (Cortes, EHA annual meeting 2011, #1019): Common drug-related adverse events for evaluable patients were nausea, QTc prolongation, vomiting, fatigue, dysgeusia, anorexia, febrile neutropenia, diarrhea, and dyspepsia. Drug-related severe adverse events in >15% of patients were febrile neutropenia and asymptomatic Grade 3 QTc prolongation. 85% of patients were evaluable for efficacy: The composite CR (CRc = CR + CRp + CRi) rate was 43% (1 CRp, 22 CRi) and PR rate was 28%. Of note, of the patients refractory to any prior therapy, 56% had CRc and 22% had PR in response to quizartinib treatment. 26% of patients were bridged to allogenic transplantation concepts.

We now report that quizartinib not only targets *FLT3* wildtype and ITD mutant kinases [24] – but also potently inhibits cellular proliferation and induces apoptosis of cells expressing a broad range of other clinically relevant class III (mutant) RTK isoforms associated with various diseases, including pediatric and adult leukemia (*FLT3 and KIT*), GIST, seminoma and melanoma (*KIT*), as well as myeloproliferative neoplasms associated with eosinophilia (*PDGFRA*). Quizartinib-responsive mutations were thereby detected in the juxtamembrane (ITDs), but also the tyrosine kinase domains (TKD I - K663Q, TKD II - D835Y) of *FLT3*, as well as in the *KIT* JM- (V560G) and TK-domains (D816Y). However, as with all tyrosine kinase inhibitors, quizartinib has a distinct potency profile against different autoactivating mutant RTK isoforms - and some mutations in the TKD of *FLT3* (D835V, B1-sheet ITDs) and *KIT* (D816V) proved to be resistant towards quizartinib. Resistancy of *FLT3* ITD mutations located in the beta-1 sheet of the first TKD has previously been shown for other TKI [32-34] as well. In contrast to the *BCR-ABL1* fusion transcript demonstrating resistance towards quizartinib, the *FIP1L1-PDGFRA* fusion mutation revealed extraordinary sensitivity. In CBF AML, which is frequently dependent upon KIT-mediated (gain-of-function) signal transduction, quizartinib demonstrated varying antiproliferative and cytotoxic efficacy in *in vitro* and *ex vivo* leukemia cells – in some cases within the low nanomolar range of *FLT3* ITD (JM) positive samples. However, the most prevalent *KIT* mutation in CBF AML, substituting valine for aspartic acid at codon 816 (KIT D816V), was demonstrated to be basically insensitive towards quizartinib in *in vitro* and *ex vivo* leukemia cell lines, primary myeloblasts, and in an isogenic Ba/F3 cell model – while substitution of a tyrosine residue (D816Y) retained some sensitivity to quizartinib. Therefore tyrosine kinase genotyping may become a prerequisite for clinical use of this agent.

Moreover, based on our data, we speculate that quizartinib may be a promising agent in solid tumors associated with *KIT* mutations, such as GIST or melanoma: In addition to a favorable activity against *KIT* mutant kinases expressed in GIST (and other mutant-*KIT* neoplasms), the excellent pharmacokinetics with unprecedented achievable plasma concentrations may be advantageous to target bulky solid tumor lesions that have impaired drug uptake. Thus, our data opens new avenues for clinical investigation and further testing of the efficacy of quizartinib in these settings is warranted.

It has to be noted, that IC50s in our studies were coherent in between all cell models used – but higher compared to a previous report [24]. The phenomenon was seen throughout the assays and is therefore most likely due to methodology reasons as we have illustrated with several experimental data:

While individual cell-context specific additional effects (such as additional mutations) can not be fully excluded to have obscured sensitivity profiles in some cell models, methodological differences most likely will account for most of the discrepancies observed: In contrast to previous studies using serum-depleted culture conditions (0.5% FBS), we used serum-repleted medium in all assays (10% FBS for cell lines, 20% for native blasts).

Even more, data from Zarrinkar and colleagues were based on treating refractory/relapsed AML samples [24] – in contrast, samples tested in our assays were isolated from patients with newly diagnosed disease. However, it is believed that refractory/relapsed patient samples have higher sensitivities towards TK-inhibition due to a higher addiction to the respective oncogenic (mutant) tyrosine kinase [37].

To underline our theories, we have provided substantial experimental data using serum-deprived versus serum-rich culture conditions in cell lines and native blasts derived from newly diagnosed as well as relapsed patients to treat with quizartinib in a dose dependent manner. High correlation of serum-levels and achievable IC50s was confirmed in all cell models. In addition, treating newly diagnosed versus relapsed *FLT3* ITD-positive leukemia native patient samples, a higher sensitivity profile for relapsed AML was verified. Thus, the data will need to be interpreted in this context.

## Conclusion

To summarize, our findings suggest that quizartinib is a promising agent for treatment of several hematologic and solid human neoplasms. However, due to the quizartinib-specific mutation restricted spectrum of activity, tyrosine kinase mutation screening may be required to identify patients most likely to respond to quizartinib therapy.

## Methods

### Cell lines

The CML blast crisis cell line K562 was a generous gift of Dr. Lopez, Oregon Health and Science University, Portland, OR. The *FLT3* ITD positive AML cell line MOLM14 (heterozygous for an ITD of DFREYE at amino acid position 593-598 [2]) was acquired through the Fujisaki Cell Center (Okayama, Japan). The acute myeloid leukemia cell line HL60, the *KIT* N822K positive CBF AML cell line Kasumi1 [41] and the *FLT3* ITD positive AML cell line MV4-11were obtained from the German Collection of Microorganisms and Cell Cultures (DSMZ). MV4-11 cells are hemizygous for an ITD of amino acids VDFREYEYDH at position 592-601 [42]. The interleukin 3 (IL-3)–dependent murine pro-B cell line Ba/F3, the FIP1L1-PDGFRA positive cell line EOL-1 [43], from a patient with eosinophilic leukemia following hypereosinophilic syndrome [44], and the p815 mast cell line was obtained from the American Type Culture Collection (ATCC, Manassas, VA). The murine *KIT* D814Y mutant isoform expressed by the p815 cell line [45] is homologous to the human *KIT* D816Y mutation. The human hematopoietic growth factor–dependent M-07e cell line was kindly provided by Dr. Hal Broxmeyer (Department of Microbiology and Immunology, Walther Oncology Center, Indiana University School of Medicine, Indianapolis, IN). The human HMC-1.1 mast cell line, expressing a *KIT* juxtamembrane domain mutant isoform (V560G), was provided by Dr. Butterfield (Division of Allergic Diseases, Department of Internal Medicine, Mayo Clinic, Rochester, MN). A spontaneously occurring subclone of the HMC-1.1 cell line, HMC-1.2, which has an additional mutation in the activation loop (D816V) [46], was kindly provided by Dr. Akin (Laboratory of Allergic Diseases, National Institute of Allergy and Infectious Diseases, NIH, Bethesda, MD). All cell lines were cultured in RPMI media containing 10% FBS.

M-07e cells were cultured using recombinant human granulocyte-macrophage colony stimulating factor (GM-CSF, R&D, Minneapolis, MN) as a growth supplement as previously described [47]. Negativity for mycoplasma contamination was confirmed using the pluripotent PCR Mycoplasma test Kit (AppliChem, Darmstadt, Germany). Cell lines harboring a mutant *KIT, FLT3* or *BCR-ABL1* were sequence confirmed.

The gastrointestinal stromal tumor cell lines GIST822, harboring a *KIT* exon 13 mutation (K642E), and GIST48, harboring an imatinib-sensitive V560D mutation plus a secondary imatinib-insensitive activation loop mutation (D820A) were generously provided by Dr. Kopp (University of Tübingen) [48,49].

### Site-directed mutagenesis and generation of a Ba/F3 cell line expressing *KIT* or *FLT3* isoforms

Site-directed mutagenesis and generation of Ba/F3 cell lines stably expressing mutant *KIT* D816V, D816Y, D816F, *FLT3* ITD, D835Y, D835Y, K663Q and *FLT3* wildtype were performed as previously described [50-52].

*KIT* Wildtype cDNA cloned into a pJP1563 plasmid vector was obtained from the DNASU Plasmid Repository at the Biodesign Institute of the Arizona State University (ASU). Lipofection transfection into the parental Ba/F3 cell line was performed to stably express *KIT* Wildtype by double selection for neomycin (pCMVneo plasmid), blasticidin (pJP1563 plasmid) or gentamicin (G418; all other plasmids) resistance and IL-3-independent growth. The Ba/F3 *KIT* Wildtype cell line was cultured using recombinant human stem cell factor (SCF/*KIT*Ligand, R&D, Minneapolis, MN) as a growth supplement.

### Antibodies and reagents

The small-molecule compounds quizartinib was obtained from Ambit Biosystems and dissolved in DMSO to create 10 mmol/L stock solutions and stored at −20°C.

Anti-*KIT* and anti-*FLT3* rabbit polyclonal antibodies were used at a 1:5,000 to 1:1,000 dilution. Antiphosphotyrosine p-*KIT* antibodies (Tyr568/570 and Tyr703), p-*FLT3* antibodies (Tyr 589/591) and a pan-antiphosphotyrosine antibody (clone PY20) were administered at dilutions of 1:100 to 1:2,000 (all from Cell Signaling Technology).

Infrared dye-conjugated secondary goat anti-rabbit or anti-mouse antibodies to use in a LI-COR® imaging detection system were prepared according to standard protocols (LI-COR Biosciences, Lincoln, NE).

### Isolation of bone marrow and peripheral blood mononuclear cells

Bone marrow aspirate and peripheral blood samples from patients with AML were collected in 5000 U heparin after informed consent and approval of the ethics committee of the University of Tübingen or Ulm. Mononuclear cells were isolated by Ficoll Hypaque density gradient fractionation [35]. Additional acute myeloid leukemia samples were generously provided by the German-Austrian AML Study Group (AMLSG) leukemia biobank (patient characteristics summarized in Additional file 2: Table S1). Native ex vivo blasts were cultured in DMEM media containing 20% FBS.

### Immunoblotting

Cell pellets were lysed with 100 to 150 μL of protein lysis buffer (50 mmol/L Tris, 150 mmol/L NaCl, 1% NP40, 0.25% deoxycholate with added inhibitors aprotinin, AEBSF, leupeptin, pepstatin, sodium orthovanadate, and sodium pyruvate, respectively phosphatase inhibitor cocktails „2“ and „1“ or „3“ (Sigma, St. Louis, MO). Protein from cell lysates (75 to 200 μg protein) was used for whole cell protein analysis after denaturing by Western immunoblot assays using a BioRad Criterion system (protein separation by SDS-PAGE in 3-8% or 10% polyacrylamide gels followed by electroblotting onto nitrocellulose membranes). Nonspecific binding was blocked by incubating the blots in nonfat dry milk or BSA. Primary antibodies were incubated for one hour or over night, followed by several washes of Tris-buffered saline (TBS) containing 0.005% Tween 20. The appropriate secondary antibody was applied for 30‘, followed by several washes. Antibody-reactive proteins were detected using a LI-COR Odyssey® fluorescence optical system (LI-COR Biosciences, Lincoln, NE).

### Apoptosis and cell viability assays

Induction of apoptosis upon quizartinib treatment was assayed in dilution series (0 – 5000 nM) and translocation of phosphatidylserine from the inner to the outer leaflet of the plasma membrane as an early indicator of apoptosis was analyzed using an Annexin V-based assay (Immunotech, Marseilles, France) and a FACScalibur® flow cytometer loaded with CellQuest® analysis software (BD, Heidelberg, Germany) [27,47].

A proportion of *ex vivo* leukemia blasts were not available for induction of apoptosis assays using Annexin V/PI staining due to a higher percentage of apoptotic cells in the untreated negative control population. Nevertheless, viability assays were assessed using FSC/SSC-flow cytometry experiments with a gate on the living cell population. Reduction of viable cells in the presence of quizartinib was measured 48 hours post quizartinib treatment.

### Proliferation assays

Cells were added to 96-well plates at densities of 50 000 cells per well. Quizartinib was added in dilution series (0 – 5000 nM) and proliferation was measured at 48 hours using an 2,3-bis[2-methoxy-4-nitro-5-sulfophenyl]-2H-tetrazolium-5-carboxanilide inner salt (XTT)–based assay (Sigma, MO) [27,47].

### Polymerase Chain Reaction (PCR) and Sequencing

Genomic DNA was isolated using a DNeasy® DNA purification *kit* (Qiagen, Hilden, Germany). *FLT3* mutation status was assessed by routine standard PCR techniques. *KIT* mutation status of exon 8, 9, 11, 13 and 17 was analyzed by PCR followed by bidirectional sequencing. The primer sets are as follows: KIT exon 8, sense: GCT GAG GTT TTC CAG CAC TC; KIT exon 8, antisense: AAT TGC AGT CCT TCC CCT CT; KIT exon 9, sense: AGCCAGGGCTTTTGTTTTCT; KIT exon 9, antisense: CAGAGCCTAAACATCCCCTTA; KIT exon 11, sense: CCTTTGCTGATTGGTTTCGT; KIT exon 11, antisense: AAACAAAGGAAGCCACTGGA; KIT exon 13, sense: GTTCCTGTATGGTACTGCATGCG; KIT exon 13, antisense: CAGTTTATAATCTAGCATTGCC; KIT exon 17, sense: GGTTTTCTTTTCTCCTCCAACC; KIT exon 17, antisense: GGATTTACATTATGAAAGTCACAGG.

### Data analysis

Inhibition of proliferation or the proportion of apoptotic/viable cells was assessed in dilution bar diagrams. Non-linear 4-parameter median-effect regression analysis was performed to compute IC50s using Prism® (GraphPad Software, Inc., LaJolla, CA) or MasterPlex® software (Hitachi Solutions, Tokyo, Japan).

## Abbreviations

AML: Acute myeloid leukemia; ALL: Acute lymphoid leukemia; CML: Chronic myeloid leukemia; FIP1L1: FIP1-like 1; FLT3: FMS-like tyrosine kinase 3; GIST: Gastrointestinal stromal tumor; HES: Hypereosinophilic syndrome; IC50: Concentration sufficient to achieve a 50% inhibition; IL3: Interleukin 3; ITD: Internal tandem duplication; KIT: v-kit Hardy-Zuckerman 4 feline sarcoma viral oncogene homolog; PDGFRA: Platelet-derived growth factor receptor alpha; RTK: Class III receptor tyrosine kinases; SM: Systemic mastocytosis; TKD1/2: Tyrosine kinase domain 1 resp. 2; TKI: Tyrosine kinase inhibitor; XTT: 2,3-Bis-(2-methoxy-4-nitro-5-sulfophenyl)-2H-tetrazolium-5-carboxanilid-sodium salt.

## Competing interests

The author(s) declare that they have no competing interest.

## Authors’ contributions

Dr. KMKS conceived of the design of the study, carried out experiments, analyzed and interpreted data and drafted the manuscript. FA: substantially participated in design and acquisition of experiments, analyzed data and helped drafting the manuscript. Dr. HD conceived of the study, analyzed and interpreted data and critically revised the manuscript. Dr. KD participated in the acquisition of data, analyzed and interpreted data and critically revised manuscript. Dr. MCH designed experiments, analyzed and interpreted data and critically revised manuscript. Dr. MMS conceived of the design of the study, analyzed and interpreted data and drafted the manuscript. All authors read and approved the final manuscript.

## Authors’ information

Grant support in part by the Deutsche Krebshilfe Foundation (MMS, KKS), the IZKF Program of the Medical Faculty Tübingen (MMS) and the Carreras Scholarship Program (KKS), a grant from the Leukemia and Lymphoma Society (MCH) and a Merit Review Grant from the Department of Veterans Affairs (MCH). We acknowledge support by Deutsche Forschungsgemeinschaft and Open Access Publishing Fund of Tübingen University.

## Supplementary Material

Additional file 1: Figure S1 Native FLT3 ITD positive patient blasts were treated with quizartinib in a dose-dependent manner and cultured in serum reduced (0.5%) versus serum repleted (20%) conditions. Cells were incubated for 48 hours and induction of apoptosis was measured using an annexin V-based assay. Densitiy plots for quizartinib at 10 nM are shown - estimated IC50s are provided with Table 2. Click here for file

Additional file 2: Table S1 Supplementary information on patient characteristics is available at the website of *MOLECULAR CANCER (see “Additional file *2* Table S1/Additional file*1* Figure S1”).* Click here for file

## References

[B1] Nakao M Yokota S Iwai T Kaneko H Horiike S Kashima K Sonoda Y Fujimoto T Misawa S Internal tandem duplication of the flt3 gene found in acute myeloid leukemia Leukemia 1996 10 1911 1918 8946930

[B2] Yokota S Kiyoi H Nakao M Iwai T Misawa S Okuda T Sonoda Y Abe T Kahsima K Matsuo Y Naoe T **Internal tandem duplication of the FLT3 gene is preferentially seen in acute myeloid leukemia and myelodysplastic syndrome among various hematological malignancies.** A study on a large series of patients and cell lines Leukemia 1997 11 1605 1609 10.1038/sj.leu.2400812 9324277

[B3] Yamamoto Y Kiyoi H Nakano Y Suzuki R Kodera Y Miyawaki S Asou N Kuriyama K Yagasaki F Shimazaki C Activating mutation of D835 within the activation loop of FLT3 in human hematologic malignancies Blood 2001 97 2434 2439 10.1182/blood.V97.8.2434 11290608

[B4] Ciolli S Vannucchi AM Leoni F Nozzoli C Longo G Salati A Pancrazzi A Bianchi L Gigli F Bosi A Internal tandem duplications of Flt3 gene (Flt3/ITD) predicts a poor post-remission outcome in adult patients with acute non-promyelocytic leukemia Leuk Lymphoma 2004 45 73 78 10.1080/1042819031000151851 15061200

[B5] Taketani T Taki T Sugita K Furuichi Y Ishii E Hanada R Tsuchida M Ida K Hayashi Y FLT3 mutations in the activation loop of tyrosine kinase domain are frequently found in infant ALL with MLL rearrangements and pediatric ALL with hyperdiploidy Blood 2004 103 1085 1088 14504097 10.1182/blood-2003-02-0418

[B6] Garcia-Montero AC Jara-Acevedo M Teodosio C Sanchez ML Nunez R Prados A Aldanondo I Sanchez L Dominguez M Botana LM KIT mutation in mast cells and other bone marrow hematopoietic cell lineages in systemic mast cell disorders: a prospective study of the Spanish Network on Mastocytosis (REMA) in a series of 113 patients Blood 2006 108 2366 2372 10.1182/blood-2006-04-015545 16741248

[B7] Beghini A Peterlongo P Ripamonti CB Larizza L Cairoli R Morra E Mecucci C C-kit mutations in core binding factor leukemias Blood 2000 95 726 727 10660321

[B8] Corbacioglu S Kilic M Westhoff MA Reinhardt D Fulda S Debatin KM Newly identified c-KIT receptor tyrosine kinase ITD in childhood AML induces ligand-independent growth and is responsive to a synergistic effect of imatinib and rapamycin Blood 2006 108 3504 3513 10.1182/blood-2006-05-021691 16840725

[B9] Whitman SP Ruppert AS Radmacher MD Mrozek K Paschka P Langer C Baldus CD Wen J Racke F Powell BL FLT3 D835/I836 mutations are associated with poor disease-free survival and a distinct gene-expression signature among younger adults with de novo cytogenetically normal acute myeloid leukemia lacking FLT3 internal tandem duplications Blood 2008 111 1552 1559 17940205 10.1182/blood-2007-08-107946 PMC2214747

[B10] Paschka P Marcucci G Ruppert AS Mrozek K Chen H Kittles RA Vukosavljevic T Perrotti D Vardiman JW Carroll AJ Adverse prognostic significance of KIT mutations in adult acute myeloid leukemia with inv(16) and t(8;21): a Cancer and Leukemia Group B Study J Clin Oncol 2006 24 3904 3911 10.1200/JCO.2006.06.9500 16921041

[B11] Cools J DeAngelo DJ Gotlib J Stover EH Legare RD Cortes J Kutok J Clark J Galinsky I Griffin JD A tyrosine kinase created by fusion of the PDGFRA and FIP1L1 genes as a therapeutic target of imatinib in idiopathic hypereosinophilic syndrome N Engl J Med 2003 348 1201 1214 10.1056/NEJMoa025217 12660384

[B12] Pardanani A Ketterling RP Brockman SR Flynn HC Paternoster SF Shearer BM Reeder TL Li CY Cross NC Cools J CHIC2 deletion, a surrogate for FIP1L1-PDGFRA fusion, occurs in systemic mastocytosis associated with eosinophilia and predicts response to imatinib mesylate therapy Blood 2003 102 3093 3096 10.1182/blood-2003-05-1627 12842979

[B13] Zota V Miron PM Woda BA Raza A Wang SA Eosinophilia with FIP1L1-PDGFRA fusion in a patient with chronic myelomonocytic leukemia J Clin Oncol 2008 26 2040 2041 10.1200/JCO.2007.15.3841 18421057

[B14] Crump M Hedley D Kamel-Reid S Leber B Wells R Brandwein J Buckstein R Kassis J Minden M Matthews J A randomized phase I clinical and biologic study of two schedules of sorafenib in patients with myelodysplastic syndrome or acute myeloid leukemia: a NCIC (National Cancer Institute of Canada) Clinical Trials Group Study Leuk Lymphoma 2010 51 252 260 10.3109/10428190903585286 20109071

[B15] Ravandi F Cortes JE Jones D Faderl S Garcia-Manero G Konopleva MY O’Brien S Estrov Z Borthakur G Thomas D Phase I/II study of combination therapy with sorafenib, idarubicin, and cytarabine in younger patients with acute myeloid leukemia J Clin Oncol 2010 28 1856 1862 10.1200/JCO.2009.25.4888 20212254 PMC2930809

[B16] Fiedler W Mesters R Tinnefeld H Loges S Staib P Duhrsen U Flasshove M Ottmann OG Jung W Cavalli F A phase 2 clinical study of SU5416 in patients with refractory acute myeloid leukemia Blood 2003 102 2763 2767 10.1182/blood-2002-10-2998 12843001

[B17] Fiedler W Serve H Dohner H Schwittay M Ottmann OG O’Farrell AM Bello CL Allred R Manning WC Cherrington JM A phase 1 study of SU11248 in the treatment of patients with refractory or resistant acute myeloid leukemia (AML) or not amenable to conventional therapy for the disease Blood 2005 105 986 993 15459012 10.1182/blood-2004-05-1846

[B18] Fischer T Stone RM Deangelo DJ Galinsky I Estey E Lanza C Fox E Ehninger G Feldman EJ Schiller GJ Phase IIB trial of oral Midostaurin (PKC412), the FMS-like tyrosine kinase 3 receptor (FLT3) and multi-targeted kinase inhibitor, in patients with acute myeloid leukemia and high-risk myelodysplastic syndrome with either wild-type or mutated FLT3 J Clin Oncol 2010 28 4339 4345 10.1200/JCO.2010.28.9678 20733134 PMC4135183

[B19] Knapper S Burnett AK Littlewood T Kell WJ Agrawal S Chopra R Clark R Levis MJ Small D A phase 2 trial of the FLT3 inhibitor lestaurtinib (CEP701) as first-line treatment for older patients with acute myeloid leukemia not considered fit for intensive chemotherapy Blood 2006 108 3262 3270 10.1182/blood-2006-04-015560 16857985

[B20] Levis M Ravandi F Wang ES Baer MR Perl A Coutre S Erba H Stuart RK Baccarani M Cripe LD Results from a randomized trial of salvage chemotherapy followed by lestaurtinib for patients with FLT3 mutant AML in first relapse Blood 2011 117 3294 3301 10.1182/blood-2010-08-301796 21270442 PMC3069671

[B21] Gotlib J Berube C Growney JD Chen CC George TI Williams C Kajiguchi T Ruan J Lilleberg SL Durocher JA Activity of the tyrosine kinase inhibitor PKC412 in a patient with mast cell leukemia with the D816V KIT mutation Blood 2005 106 2865 2870 10.1182/blood-2005-04-1568 15972446 PMC1895309

[B22] Paul C Sans B Suarez F Casassus P Barete S Lanternier F Grandpeix-Guyodo C Dubreuil P Palmerini F Mansfield CD Masitinib for the treatment of systemic and cutaneous mastocytosis with handicap: a phase 2a study Am J Hematol 2010 85 921 925 10.1002/ajh.21894 21108325

[B23] Verstovsek S Tefferi A Cortes J O’Brien S Garcia-Manero G Pardanani A Akin C Faderl S Manshouri T Thomas D Kantarjian H Phase II study of dasatinib in Philadelphia chromosome-negative acute and chronic myeloid diseases, including systemic mastocytosis Clin Cancer Res 2008 14 3906 3915 10.1158/1078-0432.CCR-08-0366 18559612 PMC5018899

[B24] Zarrinkar PP Gunawardane RN Cramer MD Gardner MF Brigham D Belli B Karaman MW Pratz KW Pallares G Chao Q AC220 is a uniquely potent and selective inhibitor of FLT3 for the treatment of acute myeloid leukemia (AML) Blood 2009 114 2984 2992 10.1182/blood-2009-05-222034 19654408 PMC2756206

[B25] Chao Q Sprankle KG Grotzfeld RM Lai AG Carter TA Velasco AM Gunawardane RN Cramer MD Gardner MF James J Identification of N-(5-tert-butyl-isoxazol-3-yl)-N’-{4-[7-(2-morpholin-4-yl-ethoxy)imidazo[2,1-b][1,3]benzothiazol-2-yl]phenyl}urea dihydrochloride (AC220), a uniquely potent, selective, and efficacious FMS-like tyrosine kinase-3 (FLT3) inhibitor J Med Chem 2009 52 7808 7816 10.1021/jm9007533 19754199

[B26] Mueller S Schittenhelm M Honecker F Malenke E Lauber K Wesselborg S Hartmann JT Bokemeyer C Mayer F Cell-cycle progression and response of germ cell tumors to cisplatin in vitro Int J Oncol 2006 29 471 479 16820891

[B27] Schittenhelm MM Shiraga S Schroeder A Corbin AS Griffith D Lee FY Bokemeyer C Deininger MW Druker BJ Heinrich MC Dasatinib (BMS-354825), a dual SRC/ABL kinase inhibitor, inhibits the kinase activity of wild-type, juxtamembrane, and activation loop mutant KIT isoforms associated with human malignancies Cancer Res 2006 66 473 481 10.1158/0008-5472.CAN-05-2050 16397263

[B28] Gajiwala KS Wu JC Christensen J Deshmukh GD Diehl W DiNitto JP English JM Greig MJ He YA Jacques SL KIT kinase mutants show unique mechanisms of drug resistance to imatinib and sunitinib in gastrointestinal stromal tumor patients Proc Natl Acad Sci USA 2009 106 1542 1547 10.1073/pnas.0812413106 19164557 PMC2635778

[B29] Smith CC Wang Q Chin CS Salerno S Damon LE Levis MJ Perl AE Travers KJ Wang S Hunt JP Validation of ITD mutations in FLT3 as a therapeutic target in human acute myeloid leukaemia Nature 2012 485 260 263 10.1038/nature11016 22504184 PMC3390926

[B30] Zheng R Klang K Gorin NC Small D Lack of KIT or FMS internal tandem duplications but co-expression with ligands in AML Leuk Res 2004 28 121 126 10.1016/S0145-2126(03)00184-X 14654075

[B31] Gunawardane R Rooks A Dao A Inhibition of FLT3 autophosphorylation and downstream signaling both in vitro and in vivo by AC220, a second generation potent and selective FLT3 inhibitor. Proceedings of the 101st Annual Meeting of the American Association for Cancer Research, 2010 Apr 17-21 2010 Washington, DC. Philadelphia (PA) Abstract 3619

[B32] Breitenbuecher F Markova B Kasper S Carius B Stauder T Bohmer FD Masson K Ronnstrand L Huber C Kindler T Fischer T A novel molecular mechanism of primary resistance to FLT3-kinase inhibitors in AML Blood 2009 113 4063 4073 10.1182/blood-2007-11-126664 19144992

[B33] Breitenbuecher F Schnittger S Grundler R Markova B Carius B Brecht A Duyster J Haferlach T Huber C Fischer T Identification of a novel type of ITD mutations located in nonjuxtamembrane domains of the FLT3 tyrosine kinase receptor Blood 2009 113 4074 4077 10.1182/blood-2007-11-125476 18483393

[B34] Kayser S Schlenk RF Londono MC Breitenbuecher F Wittke K Du J Groner S Spath D Krauter J Ganser A Insertion of FLT3 internal tandem duplication in the tyrosine kinase domain-1 is associated with resistance to chemotherapy and inferior outcome Blood 2009 114 2386 2392 10.1182/blood-2009-03-209999 19602710

[B35] Schittenhelm MM Kampa KM Yee KW Heinrich MC The FLT3 inhibitor tandutinib (formerly MLN518) has sequence-independent synergistic effects with cytarabine and daunorubicin Cell Cycle 2009 8 2621 2630 10.4161/cc.8.16.9355 19625780

[B36] Yee KW Schittenhelm M O’Farrell AM Town AR McGreevey L Bainbridge T Cherrington JM Heinrich MC Synergistic effect of SU11248 with cytarabine or daunorubicin on FLT3 ITD-positive leukemic cells Blood 2004 104 4202 4209 10.1182/blood-2003-10-3381 15304385

[B37] Pratz KW Sato T Murphy KM Stine A Rajkhowa T Levis M FLT3-mutant allelic burden and clinical status are predictive of response to FLT3 inhibitors in AML Blood 2010 115 1425 1432 10.1182/blood-2009-09-242859 20007803 PMC2826764

[B38] Corless CL Fletcher JA Heinrich MC Biology of gastrointestinal stromal tumors J Clin Oncol 2004 22 3813 3825 10.1200/JCO.2004.05.140 15365079

[B39] Kemmer K Corless CL Fletcher JA McGreevey L Haley A Griffith D Cummings OW Wait C Town A Heinrich MC KIT mutations are common in testicular seminomas Am J Pathol 2004 164 305 313 10.1016/S0002-9440(10)63120-3 14695343 PMC1602213

[B40] Beadling C Jacobson-Dunlop E Hodi FS Le C Warrick A Patterson J Town A Harlow A Cruz F 3rd Azar S KIT gene mutations and copy number in melanoma subtypes Clin Cancer Res 2008 14 6821 6828 10.1158/1078-0432.CCR-08-0575 18980976

[B41] Beghini A Magnani I Ripamonti CB Larizza L Amplification of a novel c-Kit activating mutation Asn(822)-Lys in the Kasumi-1 cell line: a t(8;21)-Kit mutant model for acute myeloid leukemia Hematol J 2002 3 157 163 10.1038/sj.thj.6200168 12111653

[B42] Quentmeier H Reinhardt J Zaborski M Drexler HG FLT3 mutations in acute myeloid leukemia cell lines Leukemia 2003 17 120 124 10.1038/sj.leu.2402740 12529668

[B43] Cools J Quentmeier H Huntly BJ Marynen P Griffin JD Drexler HG Gilliland DG The EOL-1 cell line as an in vitro model for the study of FIP1L1-PDGFRA-positive chronic eosinophilic leukemia Blood 2004 103 2802 2805 10.1182/blood-2003-07-2479 14630792

[B44] Saito H Bourinbaiar A Ginsburg M Minato K Ceresi E Yamada K Machover D Breard J Mathe G Establishment and characterization of a new human eosinophilic leukemia cell line Blood 1985 66 1233 1240 2415185

[B45] Tsujimura T Furitsu T Morimoto M Isozaki K Nomura S Matsuzawa Y Kitamura Y Kanakura Y Ligand-independent activation of c-kit receptor tyrosine kinase in a murine mastocytoma cell line P-815 generated by a point mutation Blood 1994 83 2619 2626 7513208

[B46] Furitsu T Tsujimura T Tono T Ikeda H Kitayama H Koshimizu U Sugahara H Butterfield JH Ashman LK Kanayama Y Identification of mutations in the coding sequence of the proto-oncogene c-kit in a human mast cell leukemia cell line causing ligand-independent activation of c-kit product J Clin Invest 1993 92 1736 1744 10.1172/JCI116761 7691885 PMC288334

[B47] Heinrich MC Griffith DJ Druker BJ Wait CL Ott KA Zigler AJ Inhibition of c-kit receptor tyrosine kinase activity by STI 571, a selective tyrosine kinase inhibitor Blood 2000 96 925 932 10910906

[B48] Bauer S Yu LK Demetri GD Fletcher JA Heat shock protein 90 inhibition in imatinib-resistant gastrointestinal stromal tumor Cancer Res 2006 66 9153 9161 10.1158/0008-5472.CAN-06-0165 16982758

[B49] Tuveson DA Willis NA Jacks T Griffin JD Singer S Fletcher CD Fletcher JA Demetri GD STI571 inactivation of the gastrointestinal stromal tumor c-KIT oncoprotein: biological and clinical implications Oncogene 2001 20 5054 5058 10.1038/sj.onc.1204704 11526490

[B50] Yee KW O’Farrell AM Smolich BD Cherrington JM McMahon G Wait CL McGreevey LS Griffith DJ Heinrich MC SU5416 and SU5614 inhibit kinase activity of wild-type and mutant FLT3 receptor tyrosine kinase Blood 2002 100 2941 2949 10.1182/blood-2002-02-0531 12351406

[B51] Heinrich MC Hoatlin ME Zigler AJ Silvey KV Bakke AC Keeble WW Zhi Y Reifsteck CA Grompe M Brown MG DNA cross-linker-induced G2/M arrest in group C Fanconi anemia lymphoblasts reflects normal checkpoint function Blood 1998 91 275 287 9414295

[B52] Schittenhelm MM Yee KW Tyner JW McGreevey L Haley AD Town A Griffith DJ Bainbridge T Braziel RM O’Farrell AM FLT3 K663Q is a novel AML-associated oncogenic kinase: Determination of biochemical properties and sensitivity to Sunitinib (SU11248) Leukemia 2006 20 2008 2014 10.1038/sj.leu.2404374 16990784

