# Supplementary figures and images for: Quizartinib (AC220) is a potent second generation class III tyrosine kinase inhibitor that displays a distinct inhibition profile against mutant-FLT3, -PDGFRA and -KIT isoforms
Source: Mol Cancer. 2013 Mar 7;12:19. doi: 10.1186/1476-4598-12-19 (PMC3637582; doi:10.1186/1476-4598-12-19)

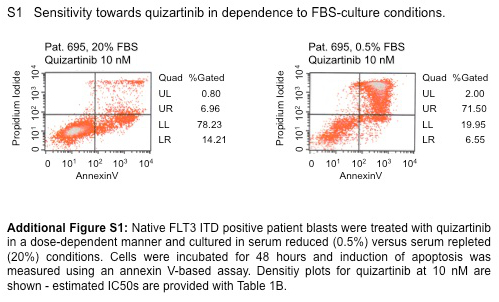

Supplement: Additional file 1: Figure S1 — Native FLT3 ITD positive patient blasts were treated with quizartinib in a dose-dependent manner and cultured in serum reduced (0.5%) versus serum repleted (20%) conditions. Cells were incubated for 48 hours and induction of apoptosis was measured using an annexin V-based assay. Densitiy plots for quizartinib at 10 nM are shown - estimated IC50s are provided with Table 2. [file 1476-4598-12-19-S1.jpeg]
