# Supplementary material for: Quizartinib (AC220) is a potent second generation class III tyrosine kinase inhibitor that displays a distinct inhibition profile against mutant-FLT3, -PDGFRA and -KIT isoforms
Source: Mol Cancer. 2013 Mar 7;12:19. doi: 10.1186/1476-4598-12-19 (PMC3637582; doi:10.1186/1476-4598-12-19)
Supplement: Additional file 2: Table S1 — Supplementary information on patient characteristics is available at the website of MOLECULAR CANCER (see “Additional file 2 Table S1/Additional file1 Figure S1”). [file 1476-4598-12-19-S2.docx]

**Supplemental table S1:**

**Patient characteristics**

**Table S1:** Patient characteristics are provided for all patients used in the activity assays (indicated with the corresponding number). Note: only patients with newly diagnosed AML were included in the studies.

N/A: not available; CBF AML: core binding factor leukemia; tAML: therapy-related AML; WT: wildtype isoform; JM: mutation in the juxtamembrane domain; beta1: mutation in the beta 1 sheet of the tyrosine kinase domain I

## 
